# Physicians in London

**Published:** 1846-05-01

**Authors:** 


*Physicians in London.*—It appears from Mr. Mitchell's Medical Dictionary that there are in London 2,157 of the medical profession. Of these, 330 are physicians, 245 surgeons, and 1,582 general practitioners.

